# Gastrodin, a Promising Natural Small Molecule for the Treatment of Central Nervous System Disorders, and Its Recent Progress in Synthesis, Pharmacology and Pharmacokinetics

**DOI:** 10.3390/ijms25179540

**Published:** 2024-09-02

**Authors:** Yanan Dai, Weikang Ban, Zhihong Yang

**Affiliations:** Institute of Medicinal Plant Development, Chinese Academy of Medical Sciences and Peking Union Medical College, Beijing 100193, China; daiyanan0604@outlook.com (Y.D.); bwk990813@163.com (W.B.)

**Keywords:** gastrodin, synthesis, pharmacology, pharmacokinetics, research progress

## Abstract

*Gastrodia elata* Blume is a traditional medicinal and food homology substance that has been used for thousands of years, is mainly distributed in China and other Asian countries, and has always been distinguished as a superior class of herbs. Gastrodin is the main active ingredient of *G. elata* Blume and has attracted increasing attention because of its extensive pharmacological activities. In addition to extraction and isolation from the original plant, gastrodin can also be obtained via chemical synthesis and biosynthesis. Gastrodin has significant pharmacological effects on the central nervous system, such as sedation and improvement of sleep. It can also improve epilepsy, neurodegenerative diseases, emotional disorders and cognitive impairment to a certain extent. Gastrodin is rapidly absorbed and widely distributed in the body and can also penetrate the blood–brain barrier. In brief, gastrodin is a promising natural small molecule with significant potential in the treatment of brain diseases. In this review, we summarised studies on the synthesis, pharmacological effects and pharmacokinetic characteristics of gastrodin, with emphasis on its effects on central nervous system disorders and the possible mechanisms, in order to find potential therapeutic applications and provide favourable information for the research and development of gastodin.

## 1. Introduction

**Figure 1:**
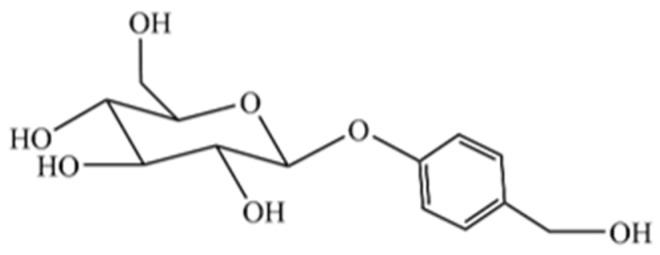
Chemical structure of gastrodin.

Gastrodin (4-hydroxybenzyl alcohol-4-*O*-*β*-D-glucopyranoside) is the main bioactive component and an important marker for quality control of the famous Chinese herb *Gastrodia elata* Blume [1,2]. *G. elata* Blume is mainly distributed in China, Korea, Japan and other places, and has always been distinguished as a superior class of herbs. This herb is a traditional medicinal and food homology substance that has been used for thousands of years and is often used to treat headaches, dizziness, epilepsy and other diseases [3,4]. The pharmacological effects of *G. elata* Blume have been continuously studied, and a recent review comprehensively elaborated on its mechanisms and functions regarding the cardiovascular system [5]. As the main active ingredient of *G. elata* Blume, gastrodin has naturally received increasing attention and research. Gastrodin (Figure 1), which has the chemical formula C_13_H_18_O_7_ and a molecular weight of 286.278 Da, is easily soluble in methanol, ethanol and water but insoluble in chloroform and ether. Gastrodin has good stability in water, methanol, ethanol and phosphate-buffered saline (PBS) solutions, and the equilibrium solubility of gastrodin in an aqueous solution decreases as the pH increases, while in organic solvents, it decreases as the polarity decreases [6]. Gastrodin has a wide range of pharmacological activities, such as sedation and hypnosis [7,8], anti-epilepsy [9,10], anti-depression [11], anti-anxiety [12], analgesia [13], blood pressure reduction [14,15], heart protection [16] and osteonecrosis prevention [17]. Among these effects, gastrodin has an outstanding effect on the central nervous system (CNS) and can be used to treat or improve epilepsy, neurodegeneration, emotional disorders, cognitive impairment and other diseases [3,4,18]. At the same time, gastrodin can reduce inflammatory reactions, improve oxidative stress injury and promote the secretion of the brain-derived neurotrophic factor (BDNF) to help the recovery of nerve function and exert good neuroprotective effects [19,20,21]. 

Gastrodin can be rapidly absorbed in the intestine and widely distributed in the body [18,22], and it can enter the brain tissue through the blood–brain barrier (BBB) [23]. As a traditional medicinal and edible homologous substance, *G. elata* Blume can be used in prescriptions and can also be eaten as food. Many studies have shown that *G. elata* Blume has no obvious toxic side effects and is safe [24,25,26]. As its main ingredient, gastrodin also shows good safety. In an acute toxicity test, oral administration or intravenous injection of gastrodin at a dosage of up to 5000 mg/kg for mice did not cause toxic reactions and death [27]. Another study also showed that intravenous administration of gastrodin at a dose of 1000 mg/kg in mice did not produce significant toxic effects [28]. 

In conclusion, gastrodin is a natural small molecule that has good development prospects. This review focuses on the pharmacological effects and related mechanisms of gastrodin in the CNS and summarises the research progress of gastrodin synthesis and pharmacokinetics in order to search for its potential therapeutic applications and provide favourable information for the research and development of gastrodin. 

## 2. Synthesis of Gastrodin

Gastrodin is the main active component of *G. elata* Blume, but its content in the rhizome is extremely low. In recent years, the resources of wild *G. elata* Blume have become fewer and fewer due to overexploitation. The traditional method used to obtain gastrodin from this herb is direct extraction and separation, which usually includes reflux extraction, ultrasound-assisted extraction and microwave-assisted extraction [29]. However, due to the limitations of gastrodin content, extraction equipment and separation and purification methods, traditional methods cannot obtain gastrodin in high yields, with high purity and at a low cost [30] and typically lead to the wastage of plant resources. In this regard, chemical synthesis and biological synthesis have gradually emerged. 

### 2.1. Chemical Synthesis of Gastrodin

**Scheme 1:**
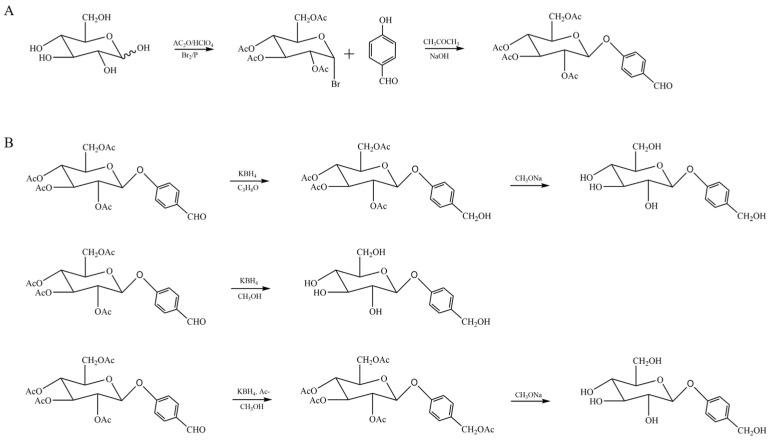
The first chemical synthesis method of gastrodin [31]. (**A**) The chemical synthesis of intermediate 4-formylphenyl 2′,3′,4′,6′-tetra-*O*-acetyl-*β*-D-glucopyranoside. (**B**) Three methods for the reduction in 4-formylphenyl 2′,3′,4′,6′-tetra-*O*-acetyl-*β*-D-glucopyranoside to gastrodin.

The synthesis of gastrodin using 2,3,4,6-tetra-*O*-acetyl-*α*-D-glucopyranosyl bromide as a glycosyl donor is the earliest chemical synthesis method (Scheme 1) [31]. After the reaction to synthesize intermediate 4-formylphenyl 2′,3′,4′,6′-tetra-*O*-acetyl-*β*-D-glucopyranoside, there are three methods to obtain gastrodin. Among them, although the third method has many steps, it is simple to operate and saves reagents. The yield of the conversion process is as high as 90%, and the total yield of glucose is up to 24%. However, as shown in Scheme 1A, the preparation of 2,3,4,6-tetra-*O*-acetyl-*α*-D-glucopyranosyl bromide involves the use of bromine and red phosphorus in the presence of perchloric acid, resulting in large quantities of highly toxic and harmful bromine and phosphorus waste pollutants that are harmful to the environment and human health. Therefore, it is unsuitable for the mass production of gastrodin because of its complex process, high cost and environmental pollution. 

**Scheme 2:**
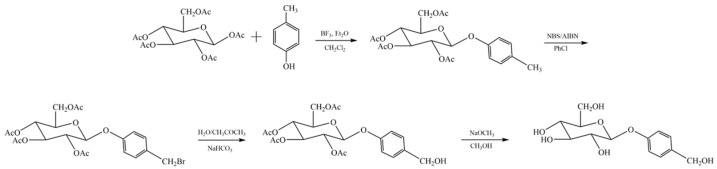
The second chemical synthesis method of gastrodin [32].

Penta-*O*-acetyl-*β*-D-glucopyranose and *p*-cresol have been used as glycosyl donors and glycosyl receptors, respectively, to synthesize gastrodin through the catalytic reaction, free radical bromination and the deprotection reaction, with a 58.1% overall yield (Scheme 2) [32]. In the first step, penta-*O*-acetyl-*β*-D-glucopyranose was treated with p-cresol to generate 4-methylphenyl 2,3,4,6-tetra-*O*-acetyl-*β*-D-glucopyranoside with a 76.3% yield. Then, the product was subjected to radical bromination to provide 4-(bromomethyl) phenyl 2,3,4,6-tetra-*O*-acetyl-*β*-D-glucopyranoside with a 91% yield. Further, the substitution reaction was performed with a solution of acetone and saturated aqueous sodium bicarbonate and led to 4-(hydroxymethyl) phenyl 2,3,4,6-tetra-*O*-acetyl-*β*-D-glucopyranoside with a 93% yield. Finally, a global deprotection reaction under Zemplen conditions achieved gastrodin with a 90% yield. This method has the advantages of operational simplicity, high overall yield, inexpensive reagents and fewer waste pollutants, possibly making it more suitable for the commercial production of gastrodin. 

Chemical synthesis is still the mainstream method for obtaining gastrodin. Although there have been many studies on improving the chemical synthesis of gastrodin from the aspects of increasing the reaction yield and reducing the reaction steps and the toxicity of reagents [32,33,34], chemical synthesis methods still have some inherent disadvantages, such as long reaction times, severe reaction conditions and lack of environmental friendliness. 

### 2.2. Biological Synthesis of Gastrodin

**Scheme 3:**
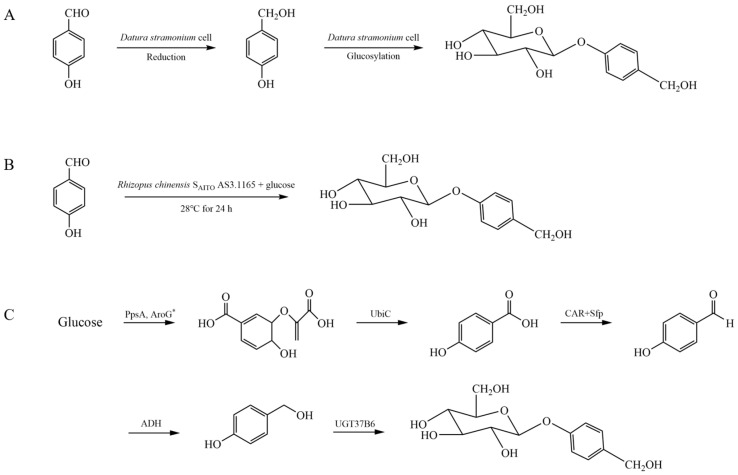
The biological synthesis methods of gastrodin [35,36,38]. (**A**) The biotransformation from *p*-hydroxybenzaldehyde to gastrodin by *Datura titular* L. cells. (**B**) The biotransformation from *p*-hydroxybenzaldehyde to gastrodin by *Rhizopus chinensis* S_AITO_ AS 3.1165. (**C**) The artificial pathway to gastrodin from glucose constructed in *E. coli*. AroG*, feedback-resistant mutant of AroG; CAR, carboxylic acid reductase; ADH, alcohol dehydrogenase; UGT, uridine sugar glycosyltransferase.

Biosynthesis is a promising way to obtain gastrodin. As shown in Scheme 3A, the suspension-cultured cells of *Datura titular* L. could reduce the precursor 4-hydroxybenzaldehyde to 4-hydroxybenzyl alcohol and then use intracellular glycosyltransferase to glycosylate it to produce gastrodin [35]. Microbial transformation has become another perspective method for producing gastrodin. *Rhizopus chinensis* S_AITO_ AS 3.1165, screened from approximately 50 fungal and bacterial strains, has the ability to bioconvert *p*-hydroxybenzaldehyde into gastrodin (Scheme 3B) [36]. Meanwhile, *Aspergillus foetidus* and *Penicillium cyclopium* AS 3.4513 could synthesize gastrodin from *p*-hydroxybenzyl alcohol (HBA) [37]. The highest yield of gastrodin was 36 mg/L for *A. foetidus* ZU-G1 and 65 mg/L for *P. cyclopium* AS 3.4513 under the respective development conditions over 6 days. Therefore, *P. cyclopium* AS 3.4513 may be the potential biocatalyst for HBA glycosylation and can be applied to the chemical synthesis process and industrial production. In addition, a heterogenic pathway for gastrodin synthesis has also been discovered (Scheme 3C). *Escherichia coli* with a *Nocardia* carboxylic acid reductase (CAR), an endogenous alcohol dehydrogenase and a *Rhodiola*-derived uridine sugar glycosyltransferase 73B6 (UGT73B6) can transform 4-hydroxybenzoic acid into gastrodin [38]. Finally, using glucose as the raw material, the engineered *E. coli* could produce 545 mg/L gastrodin in 48 h. 

The biological synthesis of gastrodin is highly reactive and can reduce energy and harmful reagent consumption, generating less waste and fewer toxic side products. However, these methods still have some problems, such as low enzyme selectivity and activity, the bacterial metabolic disorder caused by genetic modification and the unknown feasibility of mass production. Therefore, more in-depth and detailed research is needed to improve the biosynthesis methods in order to achieve high-yield and high-purity production of gastrodin. 

## 3. Pharmacological Effects of Gastrodin in the CNS

**Figure 2:**
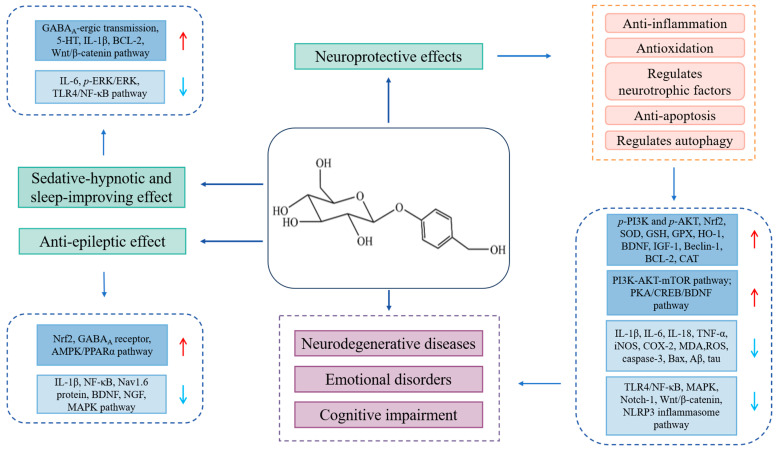
Pharmacological activities and molecular mechanisms of gastrodin in the central nervous system. The red arrows indicate up-regulation of expression, and blue arrows indicate down-regulation of expression. GABA, *γ*-aminobutyric acid; 5-HT, 5-hydroxytryptamine; IL-1β, interleukin-1beta; BCL-2, B-cell lymphoma-2; IL-6, interleukin-6; ERK, extracellular regulated protein kinases; TLR4, toll-like receptor 4; NF-κB, nuclear factor kappa-B; Nrf2, nuclear factor erythroid 2-related factor 2; AMPK, adenosine 5′-monophosphate-activated protein kinase; PPARα, peroxisome proliferators-activated receptor α; BDNF, brain-derived neurotrophic factor; NGF, nerve growth factor; MAPK, mitogen-activated protein kinase; AKT, protein kinase B; SOD, superoxide dismutase; GSH, glutathione; GPX, glutathione peroxidase; HO-1, homoxygenase-1; IGF-1, insulin-like growth factor-1; Bax, BCL-2-associated X protein; CAT, catalase; mTOR, mammalian target of rapamycin; PKA, protein kinase A; CREB, cAMP-response element binding protein; IL-18, interleukin-18; TNF-α, tumor necrosis factor-alpha; iNOS, nitric oxide synthase; COX-2, cyclooxygenase-2; MDA, malondialdehyde; ROS, reactive oxygen species; Aβ, amyloid-beta protein; NLRP3, NOD-like receptor thermal protein domain associated protein 3.

Gastrodin has a wide range of pharmacological effects involving multiple mechanisms of action in the CNS (Figure 2). Gastrodin can exert its sedative-hypnotic and anti-epileptic effects by regulating multiple signalling pathways. It can also play a neuroprotective role via anti-inflammation, antioxidation and neurotrophic factor regulation. At the same time, gastrodin can treat or improve neurodegenerative diseases, emotional disorders and cognitive impairment to some extent. Further understanding of the bioactivity and mechanisms of gastrodin in the CNS will be beneficial to its future clinical application and formulation development. 

### 3.1. Sedative-Hypnotic and Sleep-Improving Effects

Gastrodin has obvious sedative and hypnotic effects. Gastrodin can significantly inhibit autonomous activities, shorten the time required for falling asleep and play a synergistic role with sodium pentobarbital in mice [8]. Another study has also reported that gastrodin injection can significantly reduce the autonomic activities of mice, increase the number of sleep of mice and shorten the sleep latency of mice induced by a threshold dose of pentobarbital sodium and speculated that the mechanism may be related to GABA-ergic systems [39]. 

Gastrodin also has the effect of improving sleep. A study has shown that gastrodin could increase the content of 5-hydroxytryptamine (5-HT) in the brain tissue of *p*-chlorophenylalanine (PCPA)-treated mice with insomnia and regulate the expression of interleukin-6 (IL-6), interleukin-1beta (IL-1β), B-cell lymphoma-2 (BCL-2) protein and the p-extracellular regulated protein kinases (p-ERK)/ERK ratio in the brain, improving the sleep condition of PCPA mice [40]. Gastrodin treatment can also improve sleep latency and sleep duration in REM-sleep-deprivation-induced rats, and the possible mechanism behind this is that gastrodin can improve sleep disturbance and cognitive dysfunction by regulating the toll-like receptor 4 (TLR4)/nuclear factor kappa-B (NF-κB) and Wnt/β-catenin signalling pathways [7]. 

### 3.2. Anti-Epileptic Effect

Epilepsy is a neurological disease with a complex aetiology, which can seriously affect the quality of life of patients [41]. In recent years, several studies have focused on the pharmacological mechanism of gastrodin against epilepsy (Table 1). In a model of pentylenetetrazole (PTZ)-induced seizures in zebrafish, gastrodin can upregulate the activity of antioxidant enzymes and the expression of oxidative stress-related genes in a concentration-dependent manner, significantly reduce epileptoid behaviour, prolong the latency period of epilepsy and inhibit the progression of seizures [10]. Moreover, gastrodin can inhibit abnormal synchronous discharge, regulate the inflammatory responses related to the mitogen-activated protein kinase (MAPK) signalling pathway in microglia, effectively improve the intensity of seizures, increase the latency of sleep and alleviate seizures [42]. The results of an electroencephalogram (EEG), behavioural analysis and histological staining showed that gastrodin restored the levels of BDNF and nerve growth factor (NGF), activated the adenosine 5′-monophosphate-activated protein kinase (AMPK)/peroxisome proliferators-activated receptor α (PPARα) pathway and thereby remarkably alleviated epileptic symptoms in 3-week-old Sprague–Dawley (SD) rats induced by lithium-pilocarpine [43]. GABA is an important inhibitory neurotransmitter, and the loss of GABA-mediated inhibition may be the basis of neuronal over-excitation [44]. Gastrodin could reverse the degradation of GABA_A_ receptors, inhibit epileptiform discharge and protect hippocampal neuronal damage in a seizure model induced by lithium-pilocarpine and reduce the severity of acute seizures [9]. At the same time, gastrodin treatment can reverse the expression of the Nav1.6 protein in entorhinal cortex neurons experiencing epileptic status, which suggests that the inhibition of the Nav1.6 sodium current may be a potential mechanism behind the anticonvulsant properties of gastrodin [45]. However, another study showed that gastrodin did not affect the excitatory postsynaptic potential (EPSP) in the Schaffer collateral branch of the hippocampus of epileptic rats induced by N-methyl-D-aspartate (NMDA) receptors. This finding suggests that gastrodin does not interact with ionogenic glutamate receptors to inhibit NMDA receptor-promoted seizures. Nevertheless, gastrodin still showed a protective effect against NMDA toxicity on cultured hippocampal slices [46]. In conclusion, the anti-epileptic effect of gastrodin occurs by inhibiting abnormal neuronal discharge and alleviating inflammatory responses, but the underlying mechanism remains controversial and needs deeply detailed study. 

### 3.3. Neuroprotective Effects

Gastrodin has obvious neuroprotective effects. Table 2 shows the signalling pathways involved in the neuroprotective effects of gastrodin. 

#### 3.3.1. Anti-Inflammation

Microglia play a key role in the innate immune response of the CNS [66]. Activated microglia can eliminate pathogens by producing proinflammatory cytokines, but persistent nerve inflammation may induce neurotoxicity and cause neuronal damage [67,68]. Gastrodin can improve neuroinflammation through multiple signalling pathways. In an acute ocular hypertension rat model, gastrodin can significantly decrease the expression levels of microglial cytokines and p38 MAPK phosphorylation and inhibit retinal microglia activation and microglia-mediated neuroinflammation [48]. A previous study showed that gastrodin can reduce the levels of IL-1β and tumour necrosis factor-alpha (TNF-α) and the activity of NF-κB in cells exposed to H_2_O_2_, but the protective effect of gastrodin was eliminated by silencing nuclear factor erythroid2-related factor 2 (Nrf2). It is suggested that gastrodin may inhibit the inflammatory responses of H_2_O_2_-treated cells through an Nrf2-mediated mechanism [53]. Long-term exposure to lipopolysaccharide (LPS) can induce hippocampal microglia to secrete inflammatory cytokines, and gastrodin can promote the phenotype of Arg-1^+^ microglia to protect neurons from inflammatory damage and this effect is associated with the activation of Nrf2 [47]. In LPS-stimulated mouse BV-2 microglia, gastrodin inhibited the activation of the NF-κB signalling pathway and the phosphorylation of MAPKs, thereby significantly decreasing the levels of nitric oxide synthase (iNOS), cyclooxygenase-2 (COX-2) and pro-inflammatory cytokines [19]. Meanwhile, gastrodin can reverse LPS-induced activation of microglia and astrocytes in CA1, CA2, CA3 and DG regions of the hippocampus and alleviate the levels of IL-1β, interleukin-18 (IL-18), IL-6, TNF-α and other inflammatory factors. Importantly, miR-107-3p can reverse the anti-inflammatory effect of gastrodin, suggesting that its downstream target KPNA1 may be involved in the neuroprotective effect of gastrodin on LPS-induced mice [69]. 

At the same time, gastrodin can regulate the MAPKs signalling pathway of BV-2 microglia through the Notch-1 signalling pathway and inhibit the migration of BV-2 cells, and can also effectively reduce the production and release of inflammatory factors such as interleukin-23 (IL-23) and IL-1β in activated microglia, thereby inhibiting the over-activation of microglia [49]. Further studies have shown that gastrodin acts directly on the renin angiotensin system to inhibit Notch-1 signalling and remarkably reduces the expression of TNF-α and IL-1β in LPS-induced microglia [54]. The Wnt/β-catenin signalling pathway can also be regulated by gastrodin to inhibit the expression of iNOS and TNF-α in LPS-stimulated BV-2 microglia [51]. In addition, gastrodin mediates the inflammasome signalling pathway of the NOD-like receptor thermal protein domain associated protein 3 (NLRP3), thereby increasing the expression of TNF-α, IL-1β and IL-18 and reducing the inflammatory response in the brains of rats with traumatic brain injury [50]. 

Overall, gastrodin can inhibit the expression of inflammatory factors by regulating the activation of microglia and multiple signalling pathways to effectively improve the inflammatory response and damage to the nervous system. 

#### 3.3.2. Antioxidation

Oxidative stress is caused by an imbalance between the production of reactive oxygen species (ROS) and the antioxidant capacity [70]. Gastrodin exhibits an antioxidation effect in vitro and in vivo and can reduce the damage caused by oxidative stress to some extent. Studies have shown that in the brains of lead-exposed mice, gastrodin enhanced the activities of superoxide dismutase (SOD) and total antioxidant capacity (T-AOC), promoted the expression of homoxygenase-1 (HO-1) and NQO1 and restored the nuclear translocation of Nrf2, indicating that gastrodin can exhibit antioxidant effects by regulating the Wnt/Nrf2 signalling pathway [55]. Gastrodin treatment can also regulate the Nrf2/HO-1 signalling pathway, inhibit the subarachnoid haemorrhage-induced increase in the expression of oxidative stress markers, such as malondialdehyde (MDA), 3-nitrotyrosine and 8-hydroxy-2-desoxyguanosine, and restore the level of SOD, effectively playing an antioxidant role [56]. Meanwhile, it has been shown that gastrodin can increase the level of glutathione (GSH) and the activity of glutathione peroxidase (GPX), promote the expression of GPX4, Nrf2 and HO-1 and reduce the ROS level, thereby protecting C6 cells from H_2_O_2_-induced ferroptosis and reducing oxidative damages [71]. H_2_O_2_ can inhibit the mitochondrial respiratory capacity of HT-22 cells and cause oxidative stress damage. Gastrodin treatment could increase the production of neuronal ATP, repair and maintain mitochondrial function and, ultimately, protect the cells from oxidative damage [72]. Another study has shown that gastrodin can reduce ROS production and improve oxidative stress through the CaMKII/ASK-1/p38 MAPK/p53 signalling pathway, thereby preventing glutamate-induced cell death of rat pheochromocytoma (PC12) cells [57]. Hence, regulating the expression of antioxidant proteins, such as SOD, and protecting mitochondrial function are possible mechanisms behind the antioxidant effect of gastrodin, and this effect could be due to the Nrf2 signalling pathway. 

#### 3.3.3. Regulation of Neurotrophic Factors

BDNF plays an important role in the growth, development and plasticity of neurons [73]. Therefore, the upregulation of BDNF may have a protective effect on damaged neurons. Gastrodin can promote the expression of BDNF and maintain its uniform distribution in spinal cord tissue, thereby contributing to the recovery of nerve function and protecting the injured spinal cord [21]. In lead-exposed mice, gastrodin treatment significantly upregulates the expression of NR2A and BDNF in the hippocampal tissue of mice and improves lead-induced synaptic plasticity damage [55]. It has been reported that diabetes mellitus (DM) may cause neuronal apoptosis and cognitive impairments [74]. In the hippocampus of rats with DM, the expression of BDNF was significantly inhibited, and endoplasmic reticulum stress (ERS) and neuronal apoptosis also occurred. Gastrodin treatment remarkably inhibited ERS and promoted BDNF expression, suggesting that it could improve the plasticity of hippocampal nerves in DM rats [75]. Another study also showed that the expression levels of glutamate, *p*-ERK1/2, *p*-mitogen-activated extracellular signal-regulated kinase (MEK)1/2 and BDNF in the striatal neurons of streptozotocin-induced DM rats were regulated by gastrodin, which effectively alleviated neurotoxicity in neurons and significantly improved the degeneration of striatal neurons [76]. In addition, gastrodin may also improve the nerve function of rats with traumatic brain injury (TBI) by activating the protein kinase A (PKA)/cAMP-response element-binding protein (CREB)/BDNF pathway and reducing neuronal apoptosis to exert neuroprotective effects [52]. In vitro studies showed that gastrodin significantly increased the expression of BDNF and insulin-like growth factor-1 (IGF-1) in hypoxic-ischemic cell models and inhibited the expression of pro-inflammatory factors mediated by astrocytes. This result suggests that gastrodin may play a neuroprotective role through its dual effects on astrocytes [58]. In summary, gastrodin can repair damaged neurons by regulating the expression of neurotrophic factors, mainly BDNF, to play a neuroprotective role. 

#### 3.3.4. Inhibition of Apoptosis and Autophagy

Gastrodin has an inhibitory effect on apoptosis. Caspase-3 is a key biomarker of neuronal apoptosis and an executor of apoptosis, and the expression level of cleaved caspase-3 can be significantly decreased by gastrodin treatment, suggesting its good anti-neuronal apoptosis effect [59,77]. It has been demonstrated that gastrodin can reduce the protein and mRNA levels of caspase-3 in the permanent cerebral occlusion model (MCAO) and inhibit the progression of apoptosis. This indicates that the neuroprotective effects of gastrodin may be related to the inhibition of apoptosis in the hippocampus [60]. In MCAO mice, gastrodin treatment improved nerve function, reduced infarct volume and apoptosis, promoted neurogenesis through the Wnt/β-Catenin signalling pathway and thereby provided neuroprotection against brain injury [61]. Moreover, gastrodin may have inhibitory effects on cell death and apoptosis by upregulating Beclin-1 and LC3 in the neurons of rats with post-traumatic stress disorder (PTSD), thereby improving their learning and spatial memory impairments [78]. 

Gastrodin can regulate autophagy. Gastrodin can inhibit LPS-induced autophagy and apoptosis of astrocytes by regulating the PI3K-AKT-mTOR pathway [62]. Meanwhile, gastrodin can also block the upregulation of LC3B and Beclin-1 protein induced by methamphetamine (METH) through the AKT/mTOR signalling pathway, and play a protective role in the autophagy of SH-SY5Y cells [63]. In HT-22 cells exposed to hypoxia, the ratio of *p*-PI3K and *p*-AKT obviously decreased along with excessive autophagy and autophagic homeostasis. Gastrodin could increase the ratio of *p*-PI3K and *p*-AKT and maintain the autophagic homeostasis of HT-22 cells [64]. Further, gastrodin can alleviate CoCl_2_-induced autophagic flux inhibition and the formation of autophagosomes by promoting lysosomal acidification and autophagosome-lysosome fusion in HT-22 cells [65]. 

### 3.4. Effects on Neurodegenerative Diseases

Many studies have shown that gastrodin has a good effect on neurodegenerative diseases. Table 3 summarizes the models used in some studies and the dosage of gastrodin. 

#### 3.4.1. Alzheimer’s Disease

Alzheimer’s disease (AD) is the most common and severe neurodegenerative disease in the elderly and is mainly pathologically manifested by the deposition of the amyloid-beta protein (Aβ), which can form plaques and induce the hyperphosphorylation of the tau protein [98]. Soluble Aβ_1–42_ can significantly increase the spontaneous discharge activity of entorhinal cortex (EC) neurons, while gastrodin can block Aβ_1–42_-induced inward current in EC neurons and play a neuroprotective effect on the overactivity of EC neurons in rats [79]. At the same time, the treatment combining focused ultrasound (FUS) with gastrodin reduced the amount of Aβ and the levels of tau and P-tau in the hippocampus of Aβ_1–42_-induced AD-like mouse models. It can also up-regulate the levels of AQP4, BDNF, synaptophysin (SYN) and PSD-95, enhance the waste-cleaning function and neural plasticity of the brain and alleviate memory deficit [82]. In vitro analysis revealed that gastrodin can inhibit the progression of AD. Gastrodin can regulate the expression of glucose-regulated protein 78 (GRP78) and C/EBP homologous protein (CHOP) and the adaptability to cell stress, thereby exerting a protective effect on Aβ-induced cell death [84]. It also has been found that gastrodin pretreatment of primary hippocampal neurons attenuated Aβ_1–42_-induced neurotoxicity and changes in SOD and catalase (CAT), and enhanced Nrf2 expression and ERK1/2 phosphorylation, thus, the oxidative damage of hippocampal neurons was ameliorated [85]. 

The anti-inflammatory biological activity of gastrodin also plays a role in its delay in the progression of AD. A study using Tg2576 mice as a model showed that gastrodin can reduce the activation of glial cells in the mouse cortex and hippocampus and can exert neuroprotective activity by reducing neuroinflammation and the deposition of Aβ, thereby improving memory function in mice [80]. Meanwhile, gastrodin can inhibit the release of pro-inflammatory factors and apoptosis, protect primary neural progenitor cells (NPCs) from Aβ_1–42_-induced neurotoxicity and improve hippocampal neurogenesis in Aβ_1–42_-injected C57BL/6 mice. The mechanism may be related to the regulation of the MAPK signalling pathway [81]. In addition, gastrodin can reduce D-galactose (Dgal)-induced neuroinflammation and microglial activation in mice with AD by regulating the expression of the TLR4/NF-κB signalling pathway and BDNF. It can also partially target the microbiota–gut–brain axis to improve the cognitive function of mice with AD and protect the brain from damage caused by cytokines and LPS [83]. 

Overall, gastrodin can exert a neuroprotective effect by reducing the deposition of Aβ, improving nerve inflammation and oxidative damage, alleviating the symptoms caused by AD and delaying its progression. 

#### 3.4.2. Parkinson’s Disease

Parkinson’s disease (PD) is the second most common progressive neurodegenerative disease and is usually manifested by static tremors, muscle rigidity and bradykinesia [99]. Studies have shown that gastrodin can protect substantia nigral dopaminergic neurons by inhibiting the expression of IL-1β and neuroinflammation of rotenone-induced PD rats, and also can improve the muscle rigidity and endurance of the rats with PD [86]. It has been shown that gastrodin increased levels of heme oxygenase 1 (HO-1), superoxide dismutase (SOD), glutathione (GSH) and nuclear translocation of Nrf2 in the striatum of mice with 1-methyl-4-phenyl-1,2,3,6-tetrahydropyridine (MPTP)-induced PD. These results suggest that gastrodin can protect mice with PD from oxidative stress by interrupting the ERK1/2-Nrf2 pathway [90]. Gastrodin also shows neuroprotective effects in vivo and in vitro. It can prevent dopamine depletion and inhibit apoptosis and oxidative stress by reducing reactive astrogliosis and regulating the expression of BCL-2-associated X protein (Bax), BCL-2 and caspase-3, so as to effectively reduce neuronal damages [89]. The combination of FUS and gastrodin could increase the number of dopaminergic neurons in the nigrostriatal pathway of mice with PD, enhance the anti-apoptotic ability of the striatum and promote the expression of BDNF and synapse-related proteins, thereby having neuroprotective effects on mice with PD [91]. At the same time, the DAF-2/DAF-16 signalling pathway can be regulated by gastrodin to reduce the accumulation of the α-synuclein protein and rescue dopaminergic neurons in PD models [92]. In addition, gastrodin can reduce the activity of substantia nigra pars compacta (SNc) myeloperoxidase (MPO) in rats with 6-hydroxydopamine (6-OHDA)-induced PD, reduce the levels of lipid peroxidation and the production of nitric oxide (NO) and improve the catalepsy and motor incoordination induced by 6-OHDA [88]. Moreover, gastrodin could prevent PD by reducing the expression of gap junction connexin 43 (Cx43) and inhibiting the phosphorylation of Cx43 [87]. In an in vitro study, gastrodin can induce HO-1 expression by activating the p38 MAPK/Nrf2 signalling pathway and protecting SH-SY5Y cells from oxidative cell death. Hence, gastrodin has a protective effect on some dopaminergic cells, which may be important for the treatment of PD [20]. Taken together, gastrodin can alleviate the symptoms of PD by inhibiting apoptosis and oxidative stress through the regulation of related signalling pathways and exerting a protective effect on dopaminergic neurons. 

#### 3.4.3. Others

Cerebral ischemia can cause glucose/energy failure and cellular apoptosis, which play an important role in the development of neurodegenerative diseases [100,101]. A study has shown that gastrodin has a significant protective effect on striatal injury caused by cerebral ischemia, and its mechanism may be related to the upregulation of the anti-inflammatory factor BDNF and the downregulation of pro-inflammatory factor IL-6 [93]. Gastrodin can ameliorate learning and memory deficits induced by cerebral ischemia and can promote hippocampal neurogenesis after cerebral ischemia, which is partly mediated by the PDE9-cGMP-PKG signalling pathway [95]. Gastrodin can inhibit the downstream proteins caspase-1 and IL-18, significantly suppress the expression of STAT3 and NF-κB signalling pathway and thereby effectively decrease the OGD- and MCAO-stimulated expression of NLRP3 and NLRC4 inflammasome in astrocytes. It is suggested that gastrodin may have a therapeutic effect on ischemia brain injury [96]. Another study showed that gastrodin can reduce tested neuronal injury and neurobehavioral deficiency in MCAO mice, decrease the expression of cleaved Caspase 3 and Bax and increase the expression of Bcl-2. Moreover, gastrodin can significantly increase Akt phosphorylation and Nrf2 expression. These results suggest that gastrodin can alleviate cerebral ischemic damage in mice, and the activation of the Akt/Nrf2 pathway may play a critical role [94]. 

Amyotrophic lateral sclerosis (ALS) is a progressive nervous system disease that causes motor neuron degeneration. Gastrodin can coactivate the glycogen synthase kinase 3β (GSK3β) and IGF-1 pathways, thereby reducing nerve fibre cytopathies in ALS mice [97]. Traumatic brain injury (TBI) is a risk factor for neurodegenerative diseases and may initiate molecular cascades involved in neurodegenerative processes associated with dementia [102]. Gastrodin also plays a potential neuroprotective role in TBI. Gastrodin can block microglial activation-mediated inflammation through the PKA/CREB/BDNF pathway, thereby improving neurobehavioral function after TBI [52]. Gastrodin can reduce brain tissue injury and improve the functional recovery from nerve injury in TBI rats, and the mechanism may involve the inhibition of the NLRP3 inflammasome signalling pathway [50]. 

### 3.5. Effects on Emotional Disorders

#### 3.5.1. Anti-Depression

Many studies have shown that gastrodin has an antidepressant effect. Gastrodin could inhibit ER stress and the activation of the NLRP3 inflammasome and improve learning and memory function and depressive-like behaviour in mice [103]. BDNF is associated with antidepressant effects [104]. In a study that investigated the antidepressant effects of gastrodin in a rat model of chronic unpredictable stress (CUS), gastrodin protected astrocytes by activating ERK1/2, promoted BDNF expression and reversed depressive-like behaviour in CUS rats [105]. Moreover, the level of neuropeptide Y (NPY) is associated with affective disorders, and the decreased expression of NPY may induce depressive-like behaviours [106]. A single study of PTSD induced by prolonged stress (SPS) in rats suggested that gastrodin increased the expression of NPY in the hypothalamus, attenuated the levels of norepinephrine (NE) in the hippocampus and decreased the mRNA expression of BDNF. Thus, the depression-like symptoms of rats with PTSD were reversed [107]. Furthermore, a study using network pharmacology analysed and demonstrated that the effect of gastrodin on the depressive symptoms of SPS rats may be related to the decrease in corticotropin-releasing factor (CRF) expression in the hypothalamus (PVN) and central amygdala (CeA) as well as the inhibition of neuron synthesis in locus ceruleus (LC) [108]. In summary, the antidepressant effect of gastrodin may be related to the inhibition of neuroinflammation and the regulation of the expression of neurotrophic factors. 

#### 3.5.2. Anti-Anxiety

Gastrodin has an anti-anxiety effect. In the elevated plus-maze test (EPMT) and open-field test (OFT) of rats injected with complete Freund’s adjuvant (CFA), gastrodin treatment increased the number of entries in open arms and the retention times of open arms and prolonged the time in the central region of CFA rats. It suggests that gastrodin could attenuate CFA-induced anxiety-like behaviour [109]. Studies have shown that gastrodin treatment can relieve the anxiety-like symptoms induced by valproic acid (VPA) by enhancing inhibitory nerve conduction [110]. It has also been found that rats induced with PTSD by enhanced single prolonged stress (ESPS) showed anxiety-like behaviour. The levels of IL-6 and IL-1β and the expression of iNOS and the phosphorylation of MAPK in the hippocampus increased. Gastrodin treatment, especially at higher doses (200 mg/kg daily), can reverse the above changes, suggesting that gastrodin has anti-anxiety effects [12]. 

### 3.6. Effects on Cognitive Impairment

#### 3.6.1. Vascular Dementia

Vascular dementia (VaD) is a severe cognitive impairment syndrome with cerebrovascular disease as the underlying cause, and the main clinical manifestations are cognitive and memory impairment [111,112]. Ferroptosis is involved in the development of VaD, and ferroptosis inhibition has obvious neuroprotective effects [113]. It has been demonstrated that gastrodin can inhibit ferroptosis in hippocampal neurons by activating the Nrf2/Keap1-GPx4 signalling pathway, improve hippocampal damage and alleviate cognitive dysfunction in rats with VaD [114]. The deposition of Aβ induces neuronal apoptosis, and abnormal hyperphosphorylation of the tau protein leads to neuronal dysfunction [115,116]. A study on VaD rats induced by bilateral common carotid artery occlusion (BCCAO) showed that gastrodin can play a protective role by reducing the deposition of Aβ and increasing Aβ-related protein and can inhibit apoptosis by downregulating Bax expression and upregulating BCL-2 expression. This suggests that gastrodin can alleviate BCCAO-induced cognitive deficits and hippocampal neuron damage to a certain extent [117]. A further study found that gastrodin could prevent VaD-induced nerve damage by inhibiting the deposition of Aβ and abnormal phosphorylation of tau in hippocampal neurons of VaD rats, reversing learning and working memory impairment in VaD rats [118]. At the same time, gastrodin can also improve the brain energy metabolism disorder of VaD rats and reduce the mitochondrial dysfunction caused by H_2_O_2_-induced oxidative damage, thereby improving the learning and memory ability of VaD rats and the damage to neurons; hence, gastrodin plays a neuroprotective role [72]. Additionally, gastrodin attenuates impaired autophagic flux by reducing the accumulation of p62 and the aggregation of microtubule-associated protein 1 light chain 3 (LC3). The underlying mechanism is associated with the inhibition of the Ca^2+^/CaMKII signalling pathway, which could significantly reverse cognitive deficits in VaD rats [65]. In a word, gastrodin may alleviate cognitive impairment in VaD by inhibiting ferroptosis, reducing Aβ deposition and tau hyperphosphorylation and improving mitochondrial dysfunction and autophagy. 

#### 3.6.2. Other Cognitive Impairment

The development of cognitive impairment may be related to neuroinflammation mediated by inflammatory cytokines [119]. As mentioned above, gastrodin has a protective effect on neuroinflammation, which may be the basis of gastrodin improving cognitive impairment. Studies have shown that gastrodin can protect microglia and inhibit NLRP3 inflammasome by regulating the TLR4-NF-κB-NLRP3 signalling pathway and improve cognitive dysfunction caused by LPS-induced neuroinflammation in rats [120]. At the same time, gastrodin can reduce oxidative stress, neuronal apoptosis and ROS levels in mice with perioperative neurocognitive disorder (PND), activate AMPK to promote the nuclear transposition of Nrf2 and regulate the phosphorylation of GSK-3β and tau to inhibit the activation of microglia and neuroinflammation, thereby providing significant brain protection in PND mice and improving learning and memory impairment caused by surgery [121,122]. In addition, long-term gastrodin treatment also prevented working memory deficits induced by 3,3′-iminodipropionitrile (IDPN), which may be related to the improvement of the D2 receptor, dopamine (DA) and dopamine transporter (DAT) levels and dopamine turnover rate in the hippocampus of rats [123]. 

Cognitive dysfunction is also a complication of DM [124]. The results showed that gastrodin can inhibit ERS and NLRP3 inflammasome activation, increase the expression of BDNF and GLUT3 and exert neuroprotective effects on cognitive dysfunction in DM [75,103]. Gastrodin may improve the incidence of apoptotic Purkinje cells and the motor learning ability of DM rats by regulating the long-term depression (LTD) pathway [125]. Moreover, it has been demonstrated that gastrodin can restore the glucose uptake ability of neurons by inhibiting the phosphorylation of p21-activated kinase 2 (PAK2) to improve the damage to hippocampal neurons and restore the spatial learning ability of DM rats [126]. 

## 4. Pharmacokinetic Characteristics of Gastrodin

Pharmacokinetic studies can better elucidate the kinetic characteristics of drugs in vivo and explain their dynamic change rules in a comprehensive manner. Understanding the pharmacokinetic characteristics of gastrodin is conducive to further study of gastrodin. The pharmacokinetics profiles of gastrodin are shown in Table 4. 

### 4.1. Absorption

Gastrodin is absorbed quickly in the body and has an absolute bioavailability of more than 80% in rats [134]. Gastrodin could be detected in rat plasma after 5 min of intragastric administration at a dose of 100 mg/kg, and the time to maximum plasma concentration (t_max_) was 0.42 h, the half-life (t_1/2_) was 1.13 h, the maximal plasma concentration (C_max_) was 44.84 μg/mL and the area under the curve (AUC) was 57.92 μg h/mL, indicating that gastrodin was rapidly absorbed in the body [22]. A previous study used high-performance liquid chromatography (HPLC) coupled with a photodiode array detector to determine gastrodin in plasma after oral administration of a gastrodin capsule (200 mg) and showed that the t_max_ was only 0.81 h, the C_max_ was 1484.55 ng/mL and the apparent central volume of distribution (V1/F) was 180.35 L [128]. The results showed that the pharmacokinetics of gastrodin in rats and humans after intravenous injection were in accordance with the two-compartment model, while that in rats after intragastric administration was in accordance with the single-compartment model [127,133,134]. Additionally, it has been demonstrated that sodium-dependent glucose transporter 1 (STLG1) can promote gastrodin absorption in the intestine, while glucose and glucose transporter (GLT) inhibitors may affect gastrodin absorption [135]. 

### 4.2. Distribution 

Gastrodin can be widely distributed to many tissues after entering systemic circulation. Studies have shown that gastrodin was mainly distributed in rats’ kidneys, livers and lungs after 40 min of oral administration at a dose of 4 g/kg, and the peak concentrations of gastrodin were 12,584.06 ng/g, 1592.58 ng/g and 1433.08 ng/g, respectively [136]. After intragastric administration of 50 mg/kg gastrodin to rats, the concentration of gastrodin in each tissue followed the order of kidney > lung, liver, uterus > muscle, fat. At the same time, gastrodin has a low protein binding rate and does not easily accumulate in the body [137]. Gastrodin can penetrate the BBB but is concentrated in low levels in brain tissue, with peak concentrations in the brain and bile 15 min after intravenous administration [23,133,138]. As the study results have shown, gastrodin can still be found in rats’ brains after 10–20 min of intravenous administration at doses of 100 and 300 mg/kg, with C_max_ values of 1.4 and 5.2 μg/mL, respectively. However, the brain-to-blood distribution ratio (k value) of gastrodin in the brain at the two doses showed no significant difference, which may be associated with its hydrophilic characteristics [23]. 

### 4.3. Metabolism 

Gastrodin is rapidly metabolized in the body, and its main metabolite is HBA [129,139]. It has been demonstrated that gastrodin can also be metabolised into *p*-formylphenyl-*β*-D-glucopyranoside (M1), *p*-hydroxybenzonic acid (M2), *p*-formaldehydephenyl-*β*-D-glucopyranoside (M3) and *p*-hydroxybenzaldehyde (M4) in rat plasma, with C_max_ values of gastrodin > M1 > M2 > M3 > M4 > M5, but the exact metabolite pathway of gastrodin in rats has not been studied yet [139]. Another experiment studied the metabolism of gastrodin in different tissue homogenates and found that it was mainly metabolised as HBA, and the metabolic rate of each tissue followed the order of kidney > brain > liver, with values of 71.8%, 13.3% and 4.7%, respectively [140]. In addition, the intestinal flora also has some influence on the metabolism of gastrodin. Studies have shown that antibiotics can inhibit the ability of intestinal microorganisms to metabolise gastrodin to produce 4-HBA in rats, suggesting that intestinal flora plays a role in the efficacy of gastrodin [141]. 

### 4.4. Excretion 

The excretion rate of gastrodin is also very high. The experimental results have shown that the distribution t_1/2_ of gastrodin in human plasma was 3.78 h, the elimination t_1/2_ was 6.06 h and the apparent oral elimination clearance (CL/F) was 62.50 L/h [128]. Gastrodin is primarily excreted from urine as a prototype, and hepatobiliary excretion is also an important route, but it is almost not excreted from faeces and does not circulate enterohepatically in rats [23,134,137]. After intravenous administration of gastrodin at doses of 100 and 300 mg/kg, the mean residence time (MRT) in the rat blood, brain and bile were 17.4 and 23.2, 24.7 and 82.5 and 38.1 and 30.8 min, and the clearance (CL) in the rat blood was 13.3 mL/(min kg) and 10.2 mL/(min kg) [23]. 

## 5. Conclusions and Prospects

**Figure 3:**
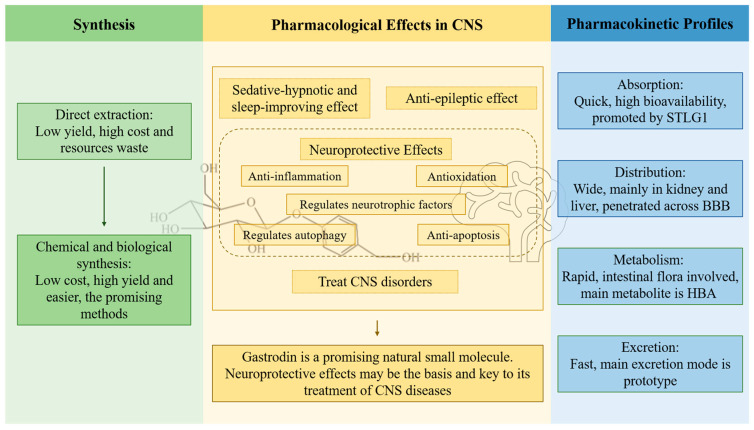
The synthesis, CNS effects and pharmacokinetics of gastrodin. CNS, central nervous system; STLG1, sodium-dependent glucose transporter 1; BBB, blood–brain barrier; HBA, p-hydroxybenzyl alcohol.

Gastrodin is the main active component of the famous traditional Chinese medicine *G. elata* Blume. This manuscript (Figure 3) reviews the synthesis and pharmacokinetics of gastrodin, with emphasis on its CNS effects and the possible mechanisms, in order to provide favourable information for further research on gastrodin. 

Gastrodin can be directly extracted from the original plant, but its direct extraction leads to low yield, high costs and resource wastage. Moreover, the resources of wild *G. elata* Blume have become fewer and fewer due to overexploitation, and the quality of cultivated *G. elata* Blume is also poor. Therefore, chemical synthesis and biotransformation methods have emerged in recent years, each of which has its own advantages and limitations. It is believed that they will be successfully applied to the mass production of gastrodin after continuous updating and improvement.

Gastrodin is a promising natural small molecule. In vitro and in vivo analyses have shown the wide range of physiological activities of gastrodin in the CNS, and it may be useful in the treatment of sleep disorders, epilepsy, AD, PD, cerebral ischemia, ALS, TBI, depression, anxiety and VaD. Among them, the neuroprotective effects of gastrodin are the most prominent. Gastrodin can ameliorate inflammatory damage in the nervous system by regulating the activation of microglia and the release of inflammatory factors, such as Il-1β, IL-6 and TNF-α. Meanwhile, it can play a protective role in oxidative stress by regulating the Nrf2 pathway and improving mitochondrial function. It can also repair damaged neurons by regulating the expression of BDNF. In addition, it can inhibit the apoptosis and autophagy of nerve cells. As reviewed above, gastrodin exhibits anti-inflammatory, antioxidant and other pharmacodynamic functions in the process of managing epilepsy, neurodegenerative diseases, emotional disorders and cognitive impairment. Obviously, the neuroprotective effects of gastrodin, such as anti-inflammation and antioxidation, may be the basis and key to the potential treatment of CNS diseases. However, elucidating the CNS activity of gastrodin based on its neuroprotective properties may not be sufficient. Meanwhile, there are few studies and data on the safety and efficacy of gastrodin. Therefore, more comprehensive and in-depth research should be conducted on the pharmacological mechanism of gastrodin in the future, and the pharmacological activity of gastrodin should also be verified using clinical trials. Although *G. elata* Blume has been used for thousands of years and is considered to be a low-toxicity Chinese medicine, it is necessary to conduct safety evaluation and toxicity experimental studies on gastrodin. 

The data from pharmacokinetic studies showed that gastrodin was rapidly absorbed, distributed, metabolised and excreted. Gastrodin can quickly penetrate the BBB and has obvious CNS effects. Moreover, intestinal flora and GLTs can affect the efficacy of gastrodin. This finding suggests that additional detailed pharmacokinetics studies on gastrodin should be carried out in the future, and scholars should focus on the interaction between gastrodin and other drugs to provide more comprehensive data and a basis for the development of gastrodin preparation and clinical application. 

In conclusion, gastrodin is a small molecule derived from natural plants with significant CNS biological activity. Its multi-target effects are highly worthy of further research and development. 

## Figures and Tables

**Table 1:** The anti-epileptic mechanism of gastrodin.

Model	Dose	Results	Reference
PTZ-induced epilepsy mice	50, 100, 200 mg/kg	Ameliorate the intensity and increase the latency of seizure, improve the EEG pattern, inhibit IL-1β expression in microglia and modulate MKP-1/MAPK signalling.	[42]
Pilocarpine-induced status epilepticus rats	10 mM/day for 9 days	Reduce behavioural and electrographic severity of SE, reverse the increase in Nav1.6 expression in mEC Layer II and terminate bursting discharge, restore excitability of mEC Layer II stellate neurons.	[45]
PTZ-induced epilepsy zebrafish	600, 800, 1000 μM	Decrease seizure-like behaviour, extend the latency period and activate antioxidant genes via upregulation of Nrf2 to eliminate ROS.	[10]
Lithium-pilocarpine induced epilepsy rats	10, 20 mg/kg	Improve EEG signalling and cognitive behaviours, alleviate neural destruction, restore the expression of BDNF and NGF, and activate AMPK/PPARα pathway in model rats.	[43]
Lithium-pilocarpine induced epilepsy rats	50 mg/kg	Reduce the seizure severity, prevent the development of established status epilepticus, inhibit the epileptiform discharge and reverse the protein degradation of GABA_A_ receptor α1 subunit.	[9]

PTZ, Pentylenetetrazole; EEG, electroencephalogram; IL-1β, Interleukin-1β; MKP-1/MAPK, MAP kinase phosphatase-1/mitogen-activated protein kinase; SE, status epilepticus; mEC, medial entorhinal cortex; Nrf2, nuclear factor erythroid 2-related factor 2; ROS, reactive oxygen species; BDNF, brain-derived neurotrophic factor; NGF, nerve growth factor; AMPK, adenosine 5‘-monophosphate-activated protein kinase; PPARα, peroxisome proliferators-activated receptor α; GABA, γ-Aminobutyric acid.

**Table 2:** Summary of signalling pathways of gastrodin’s neuroprotective effects.

Effect	Model	Dose	Signalling Pathway	Reference
Anti-inflammation	LPS-exposed male C57BL/6 mice	25, 50, 100 mg/kg/day for 10 days	Nrf2 signalling pathway	[47]
	AOH female SD rats	10, 50 mg/kg/day for 2 weeks	p38 MAPK signalling pathway	[48]
	LPS-induced SD postnatal rats	50, 100, 200 mg/kg	Notch-1 and MAPKs signalling pathway	[49]
	TBI male rats	15, 30, 60 mg/kg/day for 7 days	NLRP3 inflammasome signalling pathway	[50]
	LPS-stimulated BV2 microglia and postnatal rats	cells: 10, 20, 40, 80 μM rats: 100 mg/kg	Wnt/β-catenin signalling pathway	[51]
	TBI male SD rats and LPS-induced BV2 microglia	rats: 100 mg/kg cells: 40 μM	PKA/CREB/BDNF pathway	[52]
	LPS-stimulated microglial BV-2 cells	30, 40, 60 μM	NF-κB and MAPK signalling pathway	[19]
	H_2_O_2_-treated human SH-SY5Y cells	1–25 μM	Nrf2 signalling pathway	[53]
	LPS-activated BV-2 microglia	0.17, 0.34 mM	RAS-Notch signalling pathway	[54]
Antioxidation	Pb-exposed male ICR mice	50, 100 mg/kg/day for 4 weeks	Wnt/Nrf2 signalling pathway	[55]
	SAH male SD rats	100 mg/kg	Nrf2/HO-1 antioxidant pathway	[56]
	glutamate-induced rat PC12 cells	0.1, 1.0, 10.0 μM	CaMKII/ASK-1/p38 MAPK/p53 signalling pathway	[57]
Regulation of neurotrophic factors	TBI male SD rats and LPS-induced BV2 microglia	rats: 100 mg/kg cells: 40 μM	PKA/CREB/BDNF pathway	[52]
	OGD-prepared TNC1 cells	0.34 mM	Notch signalling pathway	[58]
Anti-apoptosis	MCAO-induced male SD rats	15, 30, 60 mg/kg	apoptotic signalling pathways	[59]
	MCAO-induced male SD rats and Wister rats	100 mg/kg	intrinsic apoptosis pathway	[60]
	MCAO-injured male C57BL/6 mice	50, 100, 200 mg/kg/day for 6 days	Wnt/β-Catenin and BCL-2 signalling pathway	[61]
Regulation of autophagy	LPS-exposed glial cells	10 μM	BCL-2 and Bax signalling pathway	[62]
	METH-treated SH-SY5Y cells	2, 4 mM	AKT/mTOR signalling pathway	[63]
	Hypoxia-exposed HT-22 cells	50, 100 μmol/L for 1.5 h	PI3K/AKT signalling pathway	[64]
	CoCl_2_-induced HT-22 cells	200 μM for 1 h	Ca^2+^/CaMKII signalling pathway	[65]

LPS, lipopolysaccharide; AOH, acute ocular hypertension; TBI, traumatic brain injury; H_2_O_2_, hydrogen peroxide; Pb, lead; SAH, subarachnoid haemorrhage; PC12, pheochromocytoma; OGD, oxygen-glucose deprivation; MCAO, middle cerebral artery occlusion; METH, methamphetamine.

**Table 3:** Model and dose of gastrodin for neurodegenerative diseases.

Model		Dose	Reference
AD	Aβ_1–42_-induced male SD rats	10 mM	[79]
	Tg2576 transgenic mice	60 mg/kg/day for 15 days	[80]
	Aβ_1–42_-injected C57BL/6 mice	60 mg/kg	[81]
	Aβ_1–42_-induced male mice	100 mg/kg/day for 15 days	[82]
	Dgal-induced mice	3, 90, 210 mg/kg/day for 9 weeks	[83]
	Aβ_1–42_-induced BV2 mouse microglial cells	10 μg/mL	[84]
	Aβ_1–42_-induced rat hippocampal neurons	1–100 μM	[85]
PD	rotenone-induced male Wistar rats	0.2 g/kg	[86]
	rotenone-induced Lewis rats	5 mg/kg	[87]
	6-OHDA-induced male Wistar rats	20, 40, 80 μg/3 μL/day for 5 days	[88]
	MPTP-induced male C57BL/6 mice	10, 30, 60 mg/kg/day for 15 days	[89]
	MPTP-induced male C57BL/6 mice	60 mg/kg/day for 2 weeks	[90]
	MPTP-induced male C57BL/6 mice	100 mg/kg/day for 19 days	[91]
	6-OHDA-induced *C. elegans*	0, 25, 50, 100, 200 μM	[92]
	MPTP-induced SH-SY5Y cells	1, 5, 25 μM	[89]
	MPP^+^-induced human dopaminergic SH-SY5Y cells	1, 5, 25 μM	[20]
Cerebral ischemia	MCAO-induced male SD rats	10 mg/kg/day for 14 days	[93]
	MCAO-induced mice	10, 50, 100 mg/kg	[94]
	BCCAO-induced mice	50, 100 mg/kg/day for 14 days	[95]
	OGD/R-exposed NSCs	0.01–1 μmol/L for 48 h	
	OGD-exposed TNA2 cells	10, 20, 50, 100 μM	[96]
ALS	ALS mice	50, 200 mg/kg	[97]
TBI	TBI male rats	15, 30, 60 mg/kg/day for 7 days	[50]
	TBI male SD rats	100 mg/kg	[52]
	LPS-induced BV2 microglia	40 μM	

AD, Alzheimer’s disease; Aβ, amyloid beta; Dgal, D-galactose; PD, Parkinson’s disease; 6-OHDA, 6-hydroxydopamine; MPTP, 1-methyl-4-phenyl-1,2,3,6-tetrahydropyridine; MPP^+^, 1-methyl-4-phenylpyridinium; MCAO, middle cerebral artery occlusion; BCCAO, bilateral common carotid artery occlusion; OGD/R, oxygen-glucose deprivation/reoxygenation; ALS, Amyotrophic lateral sclerosis; TBI, traumatic brain injury.

**Table 4:** Pharmacokinetics profiles of gastrodin.

Species	Mode of Administration	Dose	C_max _ (μg/mL)	AUC (μg h/mL)	T_1/2_ (h)	Reference
Human	iv.	600 mg	94.66 ± 23.52	391.02 ± 81.88	4.16 ± 0.82	[127]
	po.	200 mg	1.48 ± 0.28	7.21 ± 3.47	6.06 ± 3.20	[128]
Dog	ig.	40 mg/kg	23.00 ± 4.39	121.75 ± 21.79	4.80 ± 2.26	[129]
Rat	in.	50 mg/kg	-	3.70 ± 0.54	-	[130]
	iv.	50 mg/kg	-	43.30 ± 9.64	-	
	iv.	20 mg/kg	75.6 ± 6.63	22.3 ± 2.57	1.14 ± 0.380	[131]
	ig.	40 mg/kg	21.7 ± 6.90	18.1 ± 5.63	2.81 ± 2.25	
	ig.	100 mg/kg	44.84 ± 14.51	57.92 ± 11.94	1.13 ± 0.06	[22]
	ig.	40 mg/kg	21.74 ± 6.90	18.14 ± 5.63	2.81 ± 2.25	[132]
	iv.	20 mg/kg	75.65 ± 6.63	22.26 ± 2.57	1.14 ± 0.38	
	ig.	21 mg/kg	10.78 ± 0.95	9.45 ± 0.41	1.43 ± 0.67	[133]
	iv.	21 mg/kg	-	28.06 ± 2.39	1.30 ± 0.50	

iv., intravenous; in., intranasal delivery; po., oral administration; ig., intragastric; C_max_, maximal plasma concentration; AUC, area under the curve; T_1/2_, half-life.

## Data Availability

No new data were created.
